# Langerhans cell histiocytosis with aneurysmal bone cyst-like changes: a case-based literature review

**DOI:** 10.1007/s00381-023-06108-7

**Published:** 2023-07-31

**Authors:** Jonathan Tomonaga Mo, Morgan Angus Darrow, Julia Devi Sharma

**Affiliations:** 1 Department of Neurosurgery, UC Davis Medical Center, 3160 Folsom Blvd Suite 3900, Sacramento, CA 95816 USA; 2 Department of Pathology and Laboratory Medicine, UC Davis Medical Center, 4400 V St, Sacramento, CA 95817 USA

**Keywords:** Pediatric bone tumors, Pediatric skull lesions, Magnetic resonance imaging, Computed tomography

## Abstract

**Background:**

Langerhans cell histiocytosis (LCH) is a neoplastic transformation of myeloid precursors that commonly presents as an osteolytic lesion of the long or flat bones in children. Aneurysmal bone cysts (ABC) are benign neoplasms that frequently affect the metaphysis of long bones and the spine, often revealing a rapidly expansile lesion with fluid-fluid levels. LCH with secondary ABC-like changes is a rare condition that has only been reported five times, with two presentations in the cranium. The aim of this paper is to review the etiology, clinical and radiographic presentations, and treatment of this condition, as well as to present a novel case on the topic.

**Case description:**

We describe a 5-year-old boy with a rapidly growing head mass and eye pain resulting in a diagnosis of LCH with secondary ABC-like changes. Radiography demonstrated an expansile, lytic lesion of the left parietal bone with fluid-fluid levels. A confirmatory diagnosis was made through histopathology, demonstrating an inflammatory, histiocytic infiltrate staining positive for CD1a, CD68, CD207 (Langerin), and S-100. The lesion was surgically excised, and the patient recovered without any complications.

**Conclusion:**

We present a novel case of LCH with secondary ABC-like changes managed with surgical excision. While a radiographic workup with multiple imaging modalities is helpful for diagnosis, a thorough immunohistochemical analysis is essential as imaging characteristics are variable and nonspecific. Furthermore, surgical excision should be considered first-line treatment for lesions involving the skull in surgically accessible areas as it is curative, alleviates symptoms, and allows for histopathological diagnosis.

## Introduction

Langerhans cell histiocytosis (LCH) is a neoplastic transformation of myeloid precursors characterized by the expression of CD1a, CD68, CD207 (Langerin), and S-100 [1]. LCH presentations span from a unifocal, single-system lesion to disseminated disease and organ dysfunction [2]. It largely affects the skeletal system (accounting for 80% of cases) and is commonly found in the skull, spine, limbs, and pelvis [1, 3]. Despite the diversity in lesion number and location, LCH most commonly presents in the pediatric population as pain and tumor formation in localized bone. The incidence of LCH is 2.6 to 8.9 per million children while the incidence in adults is unclear, attributable to the relative rarity and nonspecific clinical presentation [1]. Risk factors for LCH are not well understood, however a higher incidence has been noted for individuals of Hispanic background and more recently, a novel SMAD6 risk variant has been implicated [4].

Aneurysmal bone cysts (ABC) are a benign, clonal neoplasm involving translocations of the USP6 gene, a ubiquitin-specific protease involved in a variety of processes including protein stability and degradation, cell signaling, angiogenesis, and inflammation [5]. ABCs are a primary bone lesion in 70% of cases. The remaining percentage of cases represent “ABC-like changes” secondary to a different primary bone tumor such as chondroblastoma, giant cell tumor, or osteosarcoma [5, 6]. While ABCs can involve any skeletal site, they have a predilection for the metaphysis of long bones (e.g., the femur or tibia) and the spine [6]. They are typically characterized by lytic, expansile, and lobulated lesions on radiograph. Though not pathognomonic, fluid-fluid levels are highly suggestive of ABCs. Clinical presentation frequently involves acute swelling and a palpable mass associated with pain, but lesion location and progression can vary. The mean age of diagnosis for an ABC is 10.2 years and ABC has a higher prevalence among males, with a male-to-female ratio of 1:1.16 [7].

The incidence of LCH and a secondary malignancy is approximately 2.6% in children [8]. Past reports include associations of LCH with Hodgkin’s and non-Hodgkin’s lymphoma, thyroid carcinoma, and acute lymphoblastic leukemia [1]. LCH with features of an ABC, however, is exceedingly rare, with only five reported cases in the literature and two involving the cranium. Here we present a 5-year-old patient with ABC-like changes secondary to unifocal LCH of the parietal bone.

## Methods

The objectives of this study are to present a case report and to review the existing literature on concomitant ABC and LCH. PubMed, Ovid, Embase, and Cochrane Central were queried from date of inception to May 2023.

### Case description

A 5-year-old boy of Hispanic background with no significant past medical history presented to the emergency department with a headache and three-month history of a painful, rapidly growing bump on the left side of the head. There was no history of trauma. Two weeks prior to presentation, the patient started feeling sporadic left eye pain without erythema, discharge, or vision changes. The patient’s parents also reported a recent bump on the right temple that self-resolved within three days. Physical examination revealed an immobile, spongy lesion on the left side of the head just superior to the pterion. The lesion was tender to palpation but there were no overlying skin changes or warmth. He had normal vital signs and a normal appearance other than an obvious skull deformity at the site of the lesion.

**Fig. 1:**
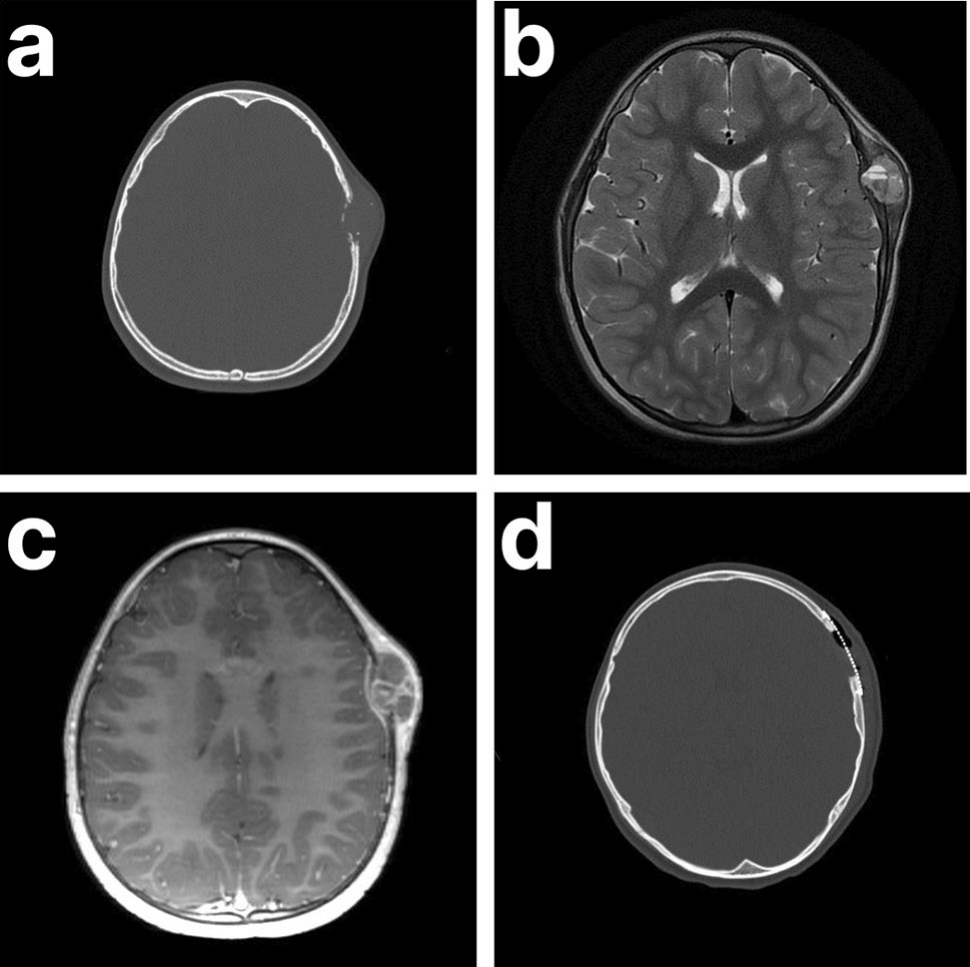
Panel **a**: CT scan shows an expansile, destructive lesion of the left parietal bone. Panel **b**: T2-weighted MRI demonstrates a well-defined, lytic lesion with internal septations and fluid-fluid levels. Panel **c**: Post-contrast T1-weighted MRI demonstrates epidural enhancement adjacent to the lesion, with no absolute evidence of dural involvement or invasion. Panel **d**: Post-operative CT demonstrating that the skull lesion was surgically resected and a titanium mesh cranioplasty was performed. *Abbreviations: CT, computed tomography; MRI, magnetic resonance imaging*

An initial ultrasound showed a non-compressible fluid collection involving soft tissues of the left cranium. A subsequent cranial X-ray was unremarkable. A computed tomography (CT) scan without contrast revealed an expansile, lytic lesion of the left parietal bone, located just posterior to the coronal suture. Heterogeneous attenuation was observed throughout the lesion (Fig. 1a). On T2-weighted magnetic resonance imaging (MRI), the lesion demonstrated fluid-fluid levels and was centered in the subgaleal space with scalloping of the outertable and suspected breach of the underlying diploic space and inner table (Fig. 1b). T1-weighted MRI demonstrated further epidural enhancement without evidence of dural invasion (Fig. 1c). The lesion measured 3.5 × 1.7 × 2.8 cm and demonstrated mild adjacent sulcal effacement. The radiographic findings favored an aneurysmal bone cyst, and less likely Langerhans cell histiocytosis, dermoid cyst, and epidermoid cyst.

**Fig. 2:**
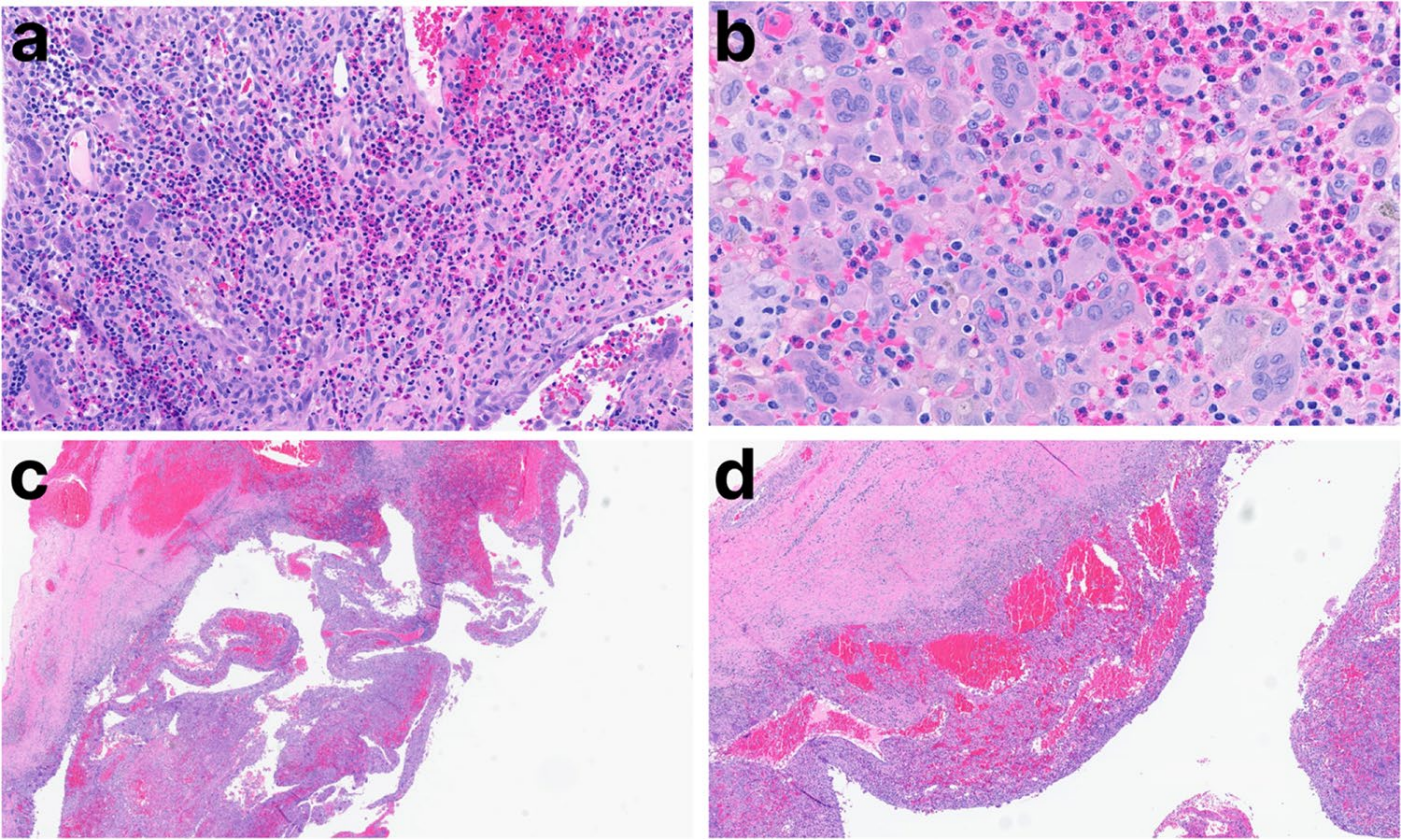
Panel **a**: Langerhans cells and multinucleate giant cells with a background of abundant eosinophils (H&E 200x). Panel **b**: Langerhans cells with characteristic nuclear grooves (so-called “coffee bean” nuclei) (H&E 400x). Panel **c**: Aneurysmal bone cyst changes with strips of cyst wall and blood (H&E 20x). Panel **d**: Aneurysmal bone cyst changes with blood-filled cystic spaces (H&E 40x). *Abbreviations: LCH, Langerhans cell histiocytosis; H&E, hematoxylin & eosin; ABC, aneurysmal bone cyst*

**Fig. 3:**
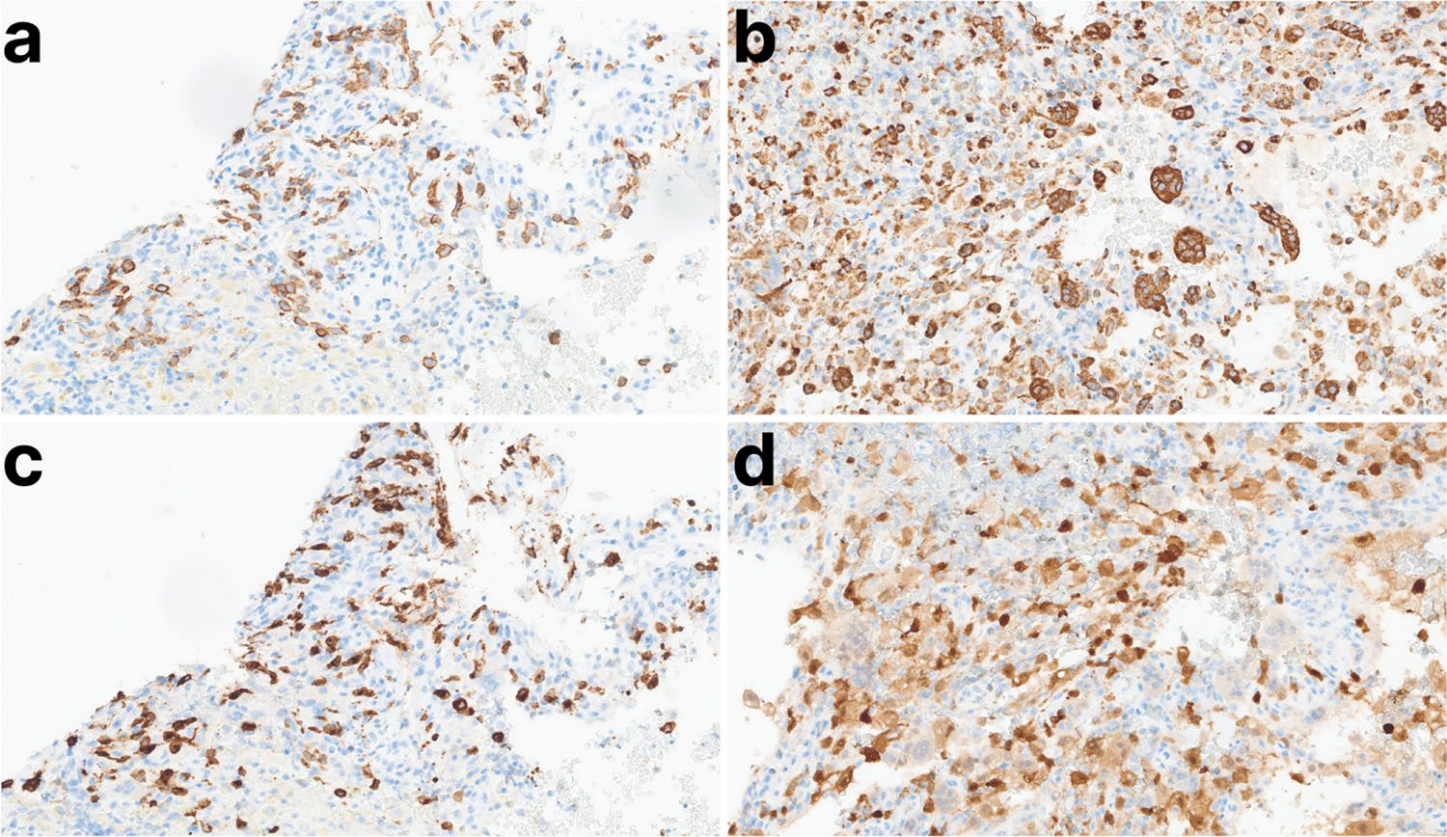
Panel **a**: CD1a IHC (200x). Panel **b**: CD68 IHC (200x). Panel **c**: CD207 (Langerin) IHC (200x). Panel **d**: S-100 IHC (200x). *Abbreviations: IHC, immunohistochemistry*

Given the patient’s significant pain and rapidly growing mass, the lesion was excised by craniectomy, and the skull repaired by titanium mesh cranioplasty (Fig. 1d). On microscopic examination, the lesion was composed of an inflammatory lymphohistiocytic infiltrate with Langerhans cells, multinucleate giant cells, and abundant eosinophils (Fig. 2a–b). By immunohistochemistry, the histiocytes expressed CD1a, CD207 (Langerin), CD68, and S-100, consistent with Langerhans cells (Fig. 3a–d). In addition to the Langerhans cell histiocytosis features, the lesion had large blood-filled cystic spaces consistent with aneurysmal bone cyst changes (Fig. 2c–d). The patient recovered without any complications and showed no evidence of recurrence six weeks post-operation.

## Discussion

LCH is a rare, neoplastic transformation of myeloid precursors that differentiate into CD1a + and CD207 + cells within lesions [1]. Defined by pathologic and constitutive activation of the MAPK signaling pathway, LCH presents variably and in all age groups, though it commonly manifests as a local osteolytic lesion of the long or flat bones in children [1–3]. Beyond the skeletal system, the skin and lungs are also commonly involved [1]. LCH is classified by both lesion site (single versus multi-system) and the number of lesions involved (local versus multi-focal), as well as involvement of risk organs such as the hematopoietic system, liver, and spleen. Multi-system involvement with cytopenia, hypersplenism, sclerosing cholangitis, or hepatic fibrosis, is a sign of poor prognosis more frequently observed in young children [1].

Radiographic findings for LCH typically show a focal, osteolytic lesion that is sharp and round, aptly described as having a “punched-out” appearance [9]. These lesions may further develop areas of surrounding sclerosis and periosteal reaction. Universal radiographic findings, however, are non-specific as other etiologies such as osteomyelitis, multiple myeloma, or plasmacytoma may demonstrate similar imaging features [10, 11]. A definitive diagnosis of LCH requires a biopsy, which shows a clonal, neoplastic proliferation with CD1a, CD207 (Langerin), CD68, and S-100 expression [1]. CD1a and CD207 are specific markers for Langerhans cells while CD68 is a general histiocytic marker and S-100 expression is abnormal in histiocytes. LCH cells have characteristic nuclear grooves, or “coffee bean” nuclei, and are typically accompanied by multinucleate giant cells and eosinophils. This is due to Langerhans cell activation and subsequent recruitment of inflammatory cells [1]. The characteristic histologic appearance, along with the positive immunohistochemical markers, is overall diagnostic for LCH. While no longer routinely performed or required for diagnosis, electron microscopy of Langerhans cells may show cytoplasmic Birbeck granules: pentalaminar, tennis-racket shaped inclusions with a central linear density and striated appearance [1].

Treatment of LCH involves risk-stratifying patients based on the severity of LCH disease and organ involvement. Unifocal, single-system lesions typically require local therapy, surgery, or observation, while systemic involvement may require more aggressive treatment with medication and radiation therapy [2, 3]. With central nervous system involvement, a one-year course of vinblastine and prednisone is the current standard of care [1]. In general, outcomes for unifocal, single-system lesions have a favorable prognosis [12]. Although survival for patients with disseminated LCH is lower (5-year survival rates of 90% for children and 70% for adults), there has been an upwards trend in survival rates with recent diagnostic and treatment advancements [1].

ABC is a benign neoplasm that commonly presents as a local lesion in children and younger adults [5]. It often affects the metaphysis of long bones and the spine, but can theoretically involve any skeletal site [6, 13]. While the pathophysiology of ABC has traditionally not been well understood, recent studies have identified USP6, a ubiquitin-specific protease, as an enzyme of interest. USP6 rearrangements exist in 65 to 70% of ABC cases and CDH11-USP6 fusion is present in an additional 30% of cases [5]. A recently revised World Health Organization Classification of Tumors of Bone (2020) recategorized “primary” and “secondary” ABC to “ABC” and “ABC-like changes,” respectively, as there exist multiple benign tumors (e.g., chondroblastoma, fibrous dysplasia, and osteosarcoma) that may demonstrate ABC-like changes without being true ABCs [5, 14].

Radiography is first-line imaging for a patient with suspected ABC. Imaging frequently shows a lytic, expansile lesion with internal septations and a sclerotic border. Fluid-fluid levels are also common but not required for diagnosis [5]. CT and MRI are helpful to confirm an ABC diagnosis and the use of multiple imaging modalities has shown improvements in sensitivity and specificity [15]. MRI may better identify fluid-fluid levels with contrasting signal intensity and can more clearly elucidate peripheral or septal enhancement. Furthermore, CT is helpful for identifying areas of cortical destruction and periosteal reaction [5]. The described imaging characteristics are also frequently observed in other primary bone tumors with secondary ABC-like changes, although these secondary changes can understandably present with more variable imaging and also tend to reflect features of the underlying, primary tumor [14, 16]. As such, location is an important diagnostic clue as a true ABC is not likely to affect the epiphysis (as in chondroblastoma or giant cell tumor), the diaphysis (as in fibrous dysplasia or non-ossifying fibroma), or the nearby soft tissues (more suggestive of giant cell tumor or osteosarcoma) [14, 16]. Histologically, ABCs are characterized by blood-filled, cystic spaces that are separated by cellular septa. They are surrounded with a thin shell of bone but do not have a true endothelial or epithelial lining [5]. The cysts often rupture, leaving behind strips of cyst wall. While not exclusive to ABCs, histology may also show osteoclast-like giant cells expressing receptor activator of nuclear kappa B (RANK), mononuclear and fibroblastic cells expressing RANK ligand, a collagenous extracellular matrix, and an osteoid component made up of bone matrix deposited by osteoblasts [5].

Treatment of ABC has traditionally involved curettage with or without adjunctive therapy. Wide-local excision may also be considered and less invasive treatments, such as sclerotherapy, arterial embolization, and monoclonal antibodies, have also been developed [5]. Despite the plethora of available treatments, no singular therapeutic has demonstrated superiority and treatment choice depends on patient presentation [17]. Surgical excision is typically curative and has a good prognosis, however ABCs have a spontaneous recurrence rate of approximately 19%, mostly within the first year of treatment [18]. Therefore, patients should be routinely monitored with radiography as recurrence may introduce bone deformities that are particularly impactful during pediatric development.

The presence of LCH with secondary ABC-like changes is rare. Only five cases have been reported in the literature with two localized to the cranium. As described in Table 1, LCH with secondary ABC-like changes have previously presented in the occiput, maxilla, and femur [9–12, 19]. Our patient presented with a rapidly growing mass in a localized region, as did three other presentations [9, 10, 12], however other patients presented with local erythema or pain without swelling [11, 19]. This demonstrates the importance of imaging for the accurate diagnosis of concomitant LCH and ABC-like changes. Common to our case and all others in Table 1, imaging consistently revealed osteolytic lesions with fluid-fluid levels and heterogeneous signal intensity. These imaging characteristics are likely to show in future presentations but are not guaranteed due to variations in how ABCs may present radiographically. On immunohistochemistry, four of the five patient biopsies tested positive for CD1a and S-100, with the fifth patient having unclear histological results [11]. Furthermore, each biopsy showed a histiocytic infiltrate with an inflammatory background. These general findings were consistent with our histological analysis, though we additionally tested for CD68 and CD207 (Langerin), which are additional markers helpful in this diagnosis. The treatment of this condition varied from surgical excision to systemic pharmacological therapy, likely due to differences in lesion localization, difficulty of surgical access, and presence of multi-organ involvement. All patients showed resolution of their symptoms after treatment. Two patients presented with rapidly growing skull lesions, and both underwent surgical resection. Notably, Krishnan and Lomoro et al. point out that the prevalence of concomitant LCH and ABC-like changes is likely underestimated due to prior limitations of immunohistochemical techniques and gaps in understanding for each disease [9, 12]. Recent advancements in disease understanding and diagnostic technology may help to explain why this unusual condition has been reported more frequently within the past four years.

**Table 1:** Prior cases presenting LCH with ABC-like changes

**Author**	**Age (years) /Gender at presentation**	**Lesion Location**	**Symptoms**	**Radiographic Findings**	**Histological Findings**	**Management**	**Outcome**
Roncaroli et al. [10]	2 (M)	Occiput	Rapidly growing mass	X-ray: lytic defect featuring sharp margins MRI (T1): well-circumscribed, multicystic lesion with a peripheral rim of decreased signal intensity and fluid-fluid levels MRI (T2): hypointensity of the septa and peripheral rim, indicating extracellular hemosiderin	Cyst wall contains hemosiderin-laden histiocytes, Langerhans cells, mature lymphocytes, and eosinophils. Langerhans cells immunoreactive to S-100 and CD1a	En bloc resection	Normal resolution
Krishnan [9]	8 (F)	Occiput	Regional pain, rapidly growing mass	MRI (T1/T2): expansile, destructive, and lytic mass in the midline of occipital bone with fluid-fluid levels and layering hemorrhage	Hemorrhagic cyst-like spaces lined by fibroblasts, inflammatory cells, histiocytes, and osteoclast-like giant cells. Septal infiltrate shows focal collections of atypical mononuclear histiocytes bearing nuclear features of LCH with enlarged nuclei and a prominent central nuclear groove. Strong staining with S-100 and CD1a	Craniotomy	Bacterial infection at surgical site, but later recovered without complications
Lomoro et al. [12]	1 (F)	Left femur	Rapidly growing mass	X-ray: large, multi-loculated osteolytic intraosseous lesion with well-defined borders and cortical bone thinning MRI (T1): non-homogenous signal with contrast showing septal enhancement MRI (T2): fluid-fluid levels	Langerhans cells immunoreactive to CD1a, S-100, CD163. Not reactive to CD34 and CD31	Pharmacological therapy (undefined)	Normal resolution
Davis et al. [11]	14 (M)	Femoral epiphysis (ABC + LCH) and cervical spine (LCH)	Neck pain and intermittent hip pain	Cervical X-ray: non-displaced anterior cortex fracture at C3 Cervical CT: lytic lesion in C3 with cortical disruption and soft tissue extension Cervical MRI: enhancing marrow alteration in C3 with anterior cortex breakthrough and area of enhancing soft tissue extending longitudinally over C2 and C4 Femoral head MRI: multilocular cystic lesion with fluid-fluid levels	Cervical biopsy: revealed large, vesicular nuclei with eosinophilic cytoplasm, background showed small lymphocytes and eosinophils Femoral head biopsy: unclear histological results	Pharmacological therapy with vinblastine and prednisone	Both cervical and femoral lesions showed normal resolution
Hwang et al. [19]	2 (F)	Maxilla	Periorbital swelling with erythema	CT: destructive lesion on the anterior wall of the right maxillary sinus MRI (T1): mass showed isointense signal MRI (T2): heterogeneous enhancement with contrast, invasion of inferior orbital wall and extraconal space with tissue edema and fat infiltration	Histiocytic cells immunoreactive to CD1a and S-100. Not reactive to H3 and 3G34W	Pharmacological therapy with vinblastine and prednisolone	Normal resolution

*ABC* aneurysmal bone cyst, *LCH* Langerhans cell histiocytosis, *M* male, *F* female, *CT* computed tomography, *MRI* magnetic resonance imaging

In this case report, we describe a 5-year-old with an unusual constellation of findings resulting in a diagnosis of LCH with secondary ABC-like changes. The patient presented with a rapidly growing mass on the head and intermittent eye pain without a history of trauma. Radiographic findings demonstrated an expansile, lytic lesion of the left parietal bone with heterogeneous attenuation and fluid-fluid levels. No dural invasion was noted. A confirmatory diagnosis was made through histopathology, demonstrating an inflammatory, histiocytic infiltrate with cells staining positive for CD1a, CD68, CD207 (Langerin), and S-100. Taken in conjunction with past reports of this condition, our case demonstrates the importance of a thorough radiographic and histological analysis for the accurate diagnosis of LCH with secondary ABC-like changes. Although radiography is the first line imaging modality, including an MRI and CT scan can help improve the sensitivity, specificity, and positive predictive value of this diagnosis [5, 16]. While the location and severity of lesions will dictate treatment, the non-specific symptoms of a rapidly growing mass and regional pain, along with ABC-like changes on radiograph, should raise suspicion for potential concurrence and a histological sample should be submitted for analysis. Surgical excision is curative, alleviates symptoms, and allows for a histopathological diagnosis. It should be considered as first-line treatment for lesions involving the skull in surgically accessible areas.
